# Genomic and phenotypic characterisation of fluoroquinolone resistance mechanisms in Enterobacteriaceae in Durban, South Africa

**DOI:** 10.1371/journal.pone.0178888

**Published:** 2017-06-21

**Authors:** John Osei Sekyere, Daniel Gyamfi Amoako

**Affiliations:** 1 Department of Pharmaceutics, Faculty of Pharmacy and Pharmaceutical Sciences, KNUST, Kumasi, Ghana; 2 Discipline of Pharmaceutical Sciences, School of Health Sciences, University of KwaZulu-Natal, Durban, South Africa; 3 Biomedical Resource Unit, College of Health Sciences, University of KwaZulu-Natal; Durban, South Africa; Seconda Universita degli Studi di Napoli, ITALY

## Abstract

Resistance to fluoroquinolones (FQ) is being increasingly reported and found to be mediated by efflux pumps, plasmid-mediated quinolone resistance genes (PMQR) and mutations in *gyrA*, *gyrB*, *parC* and *parE*. However, studies reporting on FQ resistance mechanisms (FQRM), particularly in Africa, are focused mostly on *Salmonella*. This study used a whole-genome-based approach to describe FQRM in forty-eight clinical Enterobacteriaceae isolates comprising of *Klebsiella pneumoniae* (n = 21), *Serratia marcescens* (n = 12), *Enterobacter spp*. (n = 10), *Citrobacter freundii* (n = 3), *Escherichia coli* (n = 1), and *Klebsiella michiganensis* (n = 1) with reduced susceptibility to FQ in Enterobacteriaceae. All the isolates exhibited exceptionally high-level resistance (MIC of 4-512mg/L) to all three FQs, which could not be reversed by carbonyl cyanide m-chlorophenyl hydrazine (CCCP), verapamil (VRP) or reserpine (RSP). PMQR genes such as *oqxAB* (n = 43), *aac(6’)-Ib-cr* (n = 28), and *qnr(S1*, *B1*, *B2*, *B9*, *B49*, *B66)* (n = 23) were identified without transposons or integrons in their immediate environments. Multiple and diverse mutations were found in *gyrA* (including S83I/Y and T/I83I/T), *gyrB*, *parC* and *parE*, which were clonally specific. There were vertical and horizontal transmission of high-level FQ resistance in Enterobacteriaceae in hospitals in Durban, South Africa, which are mediated by efflux, PMQR genes, and *gyrA*, *gyrB*, *parC* and *parE* mutations.

## 1. Introduction

Until recently, fluoroquinolones (FQ), which were made from the first quinolone called nalidixic acid (NAL), (a by-product of chloroquine synthesis) were very potent and were the most prescribed broad-spectrum antibiotic class for treating fatal bacterial infections [1,2]. They are the only antibiotic class that directly inhibits DNA synthesis/replication by inhibition of DNA gyrase (encoded by *gyrA* and *gyrB*) and topoisomerase IV (encoded by *parC* and *parE*) [1,2]. However, their increased overuse has led to increased resistance among both Gram-negative and Gram-positive bacteria, making them less effective [1,2]. Hence, they are used more in combination therapies with last line antibiotics such as carbapenems, colistin and tigecycline than as monotherapy [3,4].

Contrary to β-lactams that are hydrolysed by β-lactamases to cause resistance [5–7], resistance to FQ is largely mediated by point mutations in the quinolone resistance determining regions (QRDR) of *gyrA*, *gyrB*, *parC* and *parE* [1,8]. In addition, extrusion by intrinsic efflux pumps and horizontal acquisition of the plasmid-mediated quinolone resistance (PMQR) genes such as *qepA*, *qnr*, *oqxAB* and *aac(6’)-Ib-cr* have been also implicated in low-level resistance to FQ [1,2,9]. Thus, studies describing FQ resistance mechanisms (FQRM) largely focuses on finding the presence of PMQR genes, evaluating the effect of efflux on FQ resistance as well as determining the presence of mutations in *gyrA*, *gyrB*, *parC* and *parE* [1,10–12].

Although there are numerous studies characterising the prevalence and molecular epidemiology of FQRM, such studies are largely focused on *Salmonella enterica* and to a lesser extent, *Escherichia coli* and *Vibrio cholerae* through the use of PCR and pulsed field gel electrophoresis (PFGE)-based typing, particularly in Africa and South Africa [1,11–13]. Thus the ability to compare FQ-resistant strains between different countries in Africa is limited and a true genomic characterisation and epidemiology of FQRM, specifically among Enterobacteriaceae is scarce [1]. Due to the ability of enteric bacteria to associate in biofilms and share plasmids among themselves, it is imperative to broaden the scope of research beyond Salmonella to identify other Enterobacteriaceae that are reservoirs of FQ resistance. To our knowledge, there is no study using whole-genome sequencing to (WGS) describe FQRM in Enterobacteriaceae in South Africa and to a large extent, in Africa.

To provide a comprehensive description of FQRM among Enterobacteriaceae in Durban, South Africa, this study was undertaken using a large collection of diverse Enterobacteriaceae species with the view of providing a bedrock to facilitate comparative analysis in future studies and enhance meaningful epidemiological conclusions and resolutions. Moreover, an in-depth description of the transfer mechanisms of FQ resistance is important for the arrest and control of FQ-resistant strains in hospitals.

## 2. Results

### 2.1 MICs of CIP, NOR, and NAL with and without the inhibitors

The MICS of ciprofloxacin (CIP), norfloxacin (NOR) and nalidixic acid (NAL) were determined for all the isolates and controls both in the absence and presence of efflux pump inhibitors (EPIs) to assess the role of efflux pumps in FQs resistance. The MICs of NAL was very high (>512mg/L) for all the isolates whilst that of CIP and NOR ranged from 4 to 512mg/L, which makes all the isolates very resistant per the EUCAST (2016) breakpoints; MIC of >1 mg/L is defined as resistant (Table 1 and S1 Table) [14]. Most of the isolates had CIP and NOR MICs above 128mg/L.

**Table 1:** Results of norfloxacin (NOR) and ciprofloxacin (CIP) MIC changes upon adding carbonyl cyanide-m-chlorophenylhydrazine (CCCP), verapamil (VRP) and reserpine (RSP).

Isolate	MIC of Norfloxacin (NOR) (mg/L)^I^				MIC of Ciprofloxacin (CIP) (mg/L)			
	NOR	NOR + CCCP (Δ)	NOR + VRP (Δ)	NOR + RSP (Δ)	CIP	CIP + CCCP (Δ)	CIP + VRP (Δ)	CIP + RSP (Δ)
***E*. *coli ATCC 25922***	**0.03**	0.03	0.03	0.03	**0.0075**	.0075	0.004	0.0075
***K*. *oxytoca ATCC 13178***	**0.25**	0.25	0.125	0.25	**0.03**	0.03	0.015	0.03
***K*. *pneumoniae***								
**C(UNN39_S3)**	**512**	512	512	64 (**8**)	**512**	512	512	128 (4)
**D(UNN40_S4)**	**>512**	512	512	128 (4)	**512**	512	512	128 (4)
**I(UNN45_S9)**	**8**	8	8	2 (4)	**4**	4	4	2 (2)
**J(UNN46_S10)**	**512**	512	512	128 (4)	**256**	256	256	64 (4)
**3_S2**	**256**	128 (2)	512	64 (**8**)	**256**	128 (2)	256	32 (**8**)
**12_S5**	**>512**	512	512	128 (4)	**512**	512	512	128 (4)
**13_S6**	**128**	128	128	64 (2)	**128**	128	128	32 (4)
**15_S8**	**>512**	512	>512	256 (>2)	**512**	256 (2)	512	128 (4)
**18_S10**	**256**	128 (2)	512	64 (**8**)	**256**	128 (2)	256	32 (**8**)
**20_S11**	**128**	128	128	16 (**8**)	**64**	64	64	8 (4)
**21_S12**	**512**	512	512	128 (4)	**256**	256	256	64 (4)
**29_S13**	**512**	512	512	128 (4)	**256**	256	256	64 (4)
**30_S14**	**256**	128	128	32 (4)	**256**	256	256	64 (4)
**32_S15**	**>512**	512	512	128 (4)	**512**	512	512	128 (4)
**34_S16**	**256**	128 (2)	512	64 (**8**)	**256**	128 (2)	256	32 (4)
**35_S17**	**512**	256 (2)	512	64 (**8**)	**256**	256	256	64 (4)
**36_S18**	**128**	128	128	64 (2)	**128**	128	128	32 (4)
**38_S19**	**>512**	512	>512	128 (4)	**256**	128 (2)	256	64 (4)
**47_S22**	**512**	256 (2)	512	64 (**8**)	**256**	256	256	64 (4)
**52_S26**	**128**	128	128	64 (2)	**128**	128	128	32 (4)
**53_S27**	**512**	256 (2)	512	64 (**8**)	**256**	256	256	64 (4)
***S*. *marcescens***								
**B(UNN38 _S2)**	**512**	256 (2)	512	16 (**32**)	**256**	128 (2)	128 (2)	16 (16)
**E(UNN41_S5)**	**>512**	512	512	128 (4)	**512**	256 (2)	256 (2)	64 (8)
**G(UNN43_S7)**	**512**	512	512	32 (**16**)	**256**	128 (2)	256	64 (4)
**K(UNN47_S11)**	**512**	512	512	128 (4)	**256**	256	256	64 (4)
**L(UNN48_S12)**	**256**	128 (2)	256	16 (**16**)	**256**	128 (2)	256	16 (**16**)
**7_S3**	**512**	256 (2)	64 (**8**)	16 (**32**)	**256**	256	32 (**8**)	32 (**8**)
**45_S21**	**64**	64	64	4 (4)	**32**	16 (2)	32	4 (**8**)
**56_S29**	**512**	512	512	128 (4)	**256**	256	256	64 (4)
**59_S30**	**256**	128 (2)	256	128 (2)	**128**	128	128	32 (4)
**67_S33**	**512**	256 (2)	64 (**8**)	16(**32**)	**256**	256	32 (**8**)	32 (**8**)
**68_S34**	**256**	256	256	16 (**16**)	**64**	32 (2)	64	8 (**8**)
**71_S36**	**512**	512	512	128 (4)	**256**	256	256	64 (4)
***Enterobacter species*^II^**								
**A (UNN37_S1)**	**256**	64 (4)	64 (4)	4 (**64**)	**64**	32 (2)	64	2 (**32**)
**F (UNN42_S6)**	**512**	512	512	8 (**64**)	**256**	256	256	16 (**16**)
**H (UNN44_S8)**	**512**	512	512	32 (**16**)	**256**	256	256	64 (4)
**1_S1**	**8**	8	4 (2)	2 (4)	**8**	8	8	2 (4)
**16_S9**	**512**	512	64 (**8**)	32 (**16**)	**128**	64 (2)	32 (4)	8 (**16**)
**43_S20**	**8**	8	4 (2)	2 (4)	**8**	8	8	2 (4)
**49_S24**	**32**	16 (2)	16 (2)	4 (**8**)	**32**	16 (2)	16 (2)	2 (**16**)
**55_S28**	**128**	128	128	8 (**16**)	**64**	64	64	4 (**16**)
**63_S31**	**8**	8	4 (2)	2 (4)	**8**	8	8	2 (4)
**65_S32**	**512**	512	512	128 (4)	**256**	256	256	64 (4)
***E*. *coli***								
**10_S4**	**512**	512	256 (2)	128 (4)	**512**	512	512	128 (4)
***C*. *freundii***								
**14_S7**	**512**	256 (2)	64 (**8**)	16 (**32**)	**256**	256	32 (**8**)	32 (**8**)
**48_S23**	**512**	256 (2)	64 (**8**)	16 (**32**)	**256**	256	32 (**8**)	32 (**8**)
**51_25**	**256**	256	256	16 (**16**)	**64**	32 (2)	64	8 (**8**)
***K*. *michangenesis***								
**69_S35**	**128**	64 (2)	8 (**16**)	4 (**32**)	**64**	64	8 (**8**)	4 (**16**)

^I^ MICs were interpreted per EUCAST breakpoints for 2016: CIP and NOR Resistance > 1mg/L

^II^See Table 2 for species breakdown

An MIC fold change of ≥8 was adopted as significant [15]. Hence, none of the inhibitors viz., carbonyl cyanide-m-chlorophenylhydrazine (CCCP), verapamil (VRP) and reserpine (RSP) could affect the MICs of NAL (Table 1 and S1 Table) significantly i.e. had a fold change of ≥8, albeit fold changes of 2–4 were recorded in *S*. *marcescens*, *Enterobacter spp*., *C*. *freundii* and *K*. *michiganensis*. CCCP, followed by VRP, had the least effect on the MICs of the antibiotics in all the species whilst RSP had the most effect; majority of the MIC fold changes effected by RSP were significant (Table 1 and S1 Table). RSP resulted in more significant MIC fold changes in NOR (n = 24) than in CIP (n = 16) and was the only EPI that effected a consistently significant MIC fold-change reduction in all the isolates except for NAL (S1 Table).

Among the species, *E*. *coli* had no significant MIC fold change and only RSP resulted in significant MIC fold changes in *K*. *pneumoniae;* however, both RSP and VRP resulted in significant fold changes in the remaining species. None of the inhibitors could reverse resistance to any of the antibiotics. The MICs (of either CIP and/or NOR) of only 25 isolates were significantly affected by both VRP and RSP (Table 1).

### 2.2 Species frequency and distribution of plasmid-mediated quinolone resistance (PMQR) genes

**Fig 1:**
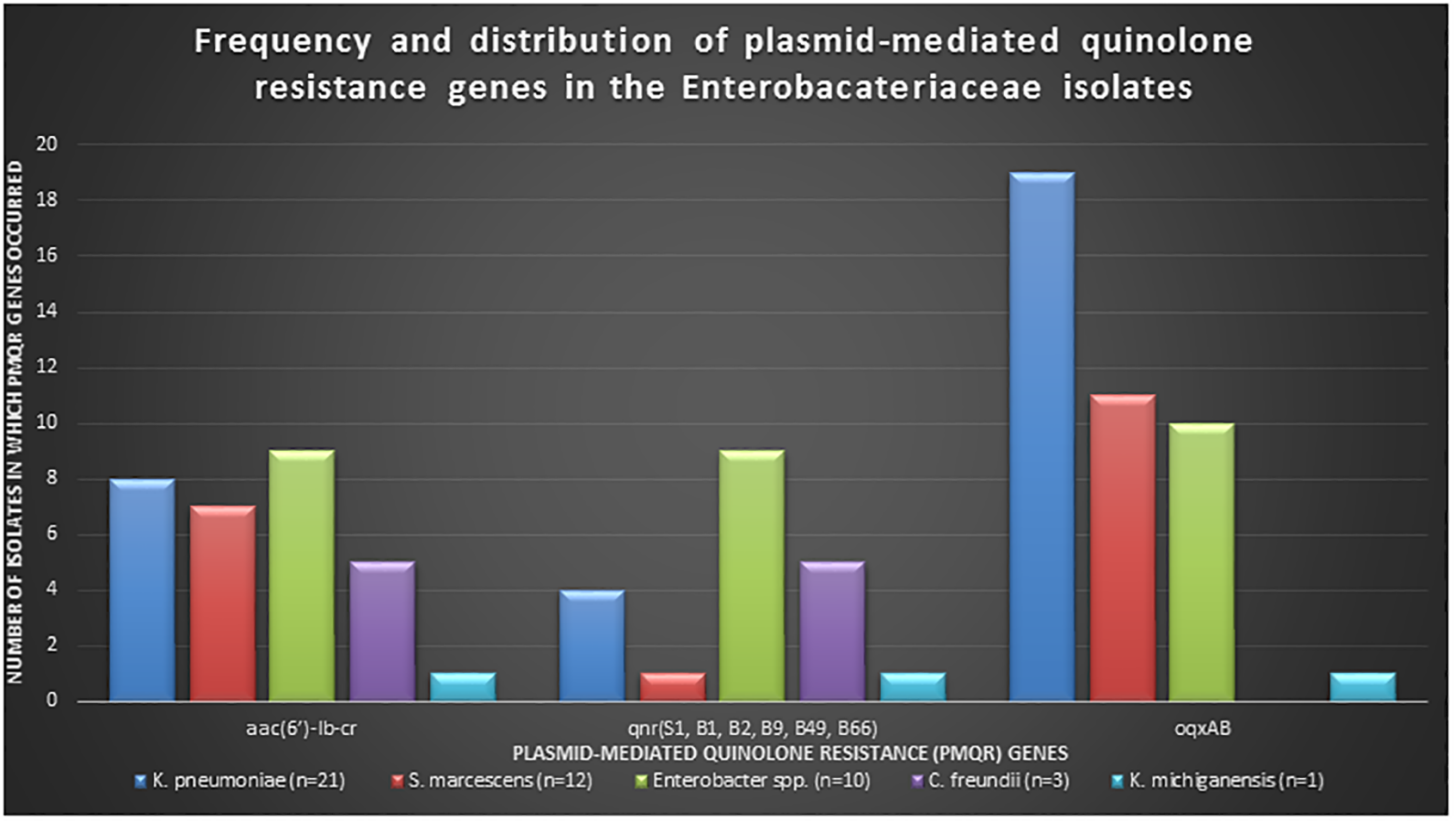
Frequency and distribution of plasmid-mediated quinolone resistance (PMQR) genes per specie. PMQR genes occurred in all isolates except E. coli. oqxAB genes were the most common occurring PMQR gene(s) followed by aac(6’)-Ib-cr and qnr genes. oqxAB was common in K. pneumoniae whilst aac(6’)-Ib-cr and qnr were dominant in Enterobacter spp.

The presence and frequency distribution of each PMQR gene was assessed in each isolate’ genome sequence. No *qepA* gene was found in any of the isolates. Moreover, no PMQR gene was found in the *E*. *coli* strain. *OqxAB*, *aac(6’)-Ib-cr*, and *qnr(S1*, *B1*, *B2*, *B9*, *B49*, *B66)*, were found in 43, 28, and 23 isolates respectively (Fig 1, Table 2). *OqxA* occurred in 20 isolates whilst *oqxB* was found in 43; hence, whilst *oqxB* alone was found in 23 isolates, *oqxA* was always found alongside *oqxB*. *OqxA* and *oqxB* occurred together in 20 isolates, 10 of which were *Enterobacter spp*., 9 were *K*. *pneumoniae* and one was *K*. *michiganensis* (Fig 1, Table 3). There was no *oqxAB* in *C*. *freundii* and no *oqxA* was present in *S*. *marcescens*. *Aac(6’)-Ib-cr* was commonest in *Enterobacter spp*. (n = 9), *K*. *pneumoniae* (n = 8), and *S*. *marcescens* (n = 7) respectively. *Qnr* genes were commonly found in *Enterobacter spp*. (n = 11 *qnr* genes), *C*. *freundii* (n = 6 *qnr* genes) and *K*. *pneumoniae* (n = 4 *qnr* genes), with *qnrS1* (n = 7), *qnrB1* (n = 6) *qnrB49* (n = 4) being the most common variants (Tables 2 and 3).

**Table 2:** Sources and fluoroquinolone/quinolone resistance mechanisms of the Enterobacteriaceae isolates.

Isolate (clone)	Specimen type	Plasmid-mediated fluoroquinolone resistance genes	Chromosomal mutations			
			*gyrA*	*gyrB*	*ParC*	*parE*
***K*. *pneumoniae*** ^III^						
53_S27 (ST101)	Urine	aac(6’)-ib-cr, OqxA, OqxB,	S83Y, D87G	D553V	S80I, N304S	NM
52_S26 (ST101)	Pus	aac(6’)-ib-cr, OqxA, OqxB, QnrS1				
47_S22 (ST1478)	Art line	aac(6’)-ib-cr, OqxA, OqxB, QnrB66	V855A	M144I, E145V, L173H, S558A, L605M, L591V, H592R, K595S, H599Q, V663I, A692T	S438D, D475E, L495Q, Q592R, V691I	N130T, N209D, S213R, S225C, A242T, I603M
38_S19 (ST101)	X^IV^	oqxB	S83Y, D87G	D553V	S80I, N304S	NM
36_S18 (ST101)	Sputum	OqxB				
35_S17(ST101)	Urine	OqxB				
34_S16(ST101)	Pus swab (Trachea)	OqxB				
32_S15(ST101)	Catheter tip	aac(6’)-ib-cr, OqxB				
30_S14(ST101)	Urine	OqxB				
29_S13(ST2017)	Abdominal swab	OqxB				
21_S12(ST2017)	Urine	oqxA, oqxB				
20_S11(ST2017)	Tracheal fluid	OqxA, OqxB				
18_S10(ST101)	urine	OqxB				
15_S8(ST101)	Pus swab (leg)	OqxB				
13_S6 (ST2016)	X	aac(6’)-ib-cr, OqxB				
12_S5(ST101)	Blood culture	oqxB				
3_S2(ST14)	Urine	Aac(6’)-ib-cr, OqxB, OqxA, QnrB1	S83Y, D87G, I54V	NM	S80I	
J(UNN46_S10(ST101))	X	aac(6’)-ib-cr, OqxB	S83Y, D87G	D553V	S438D, D475E, L495Q, Q592R, V691I	
I(UNN45_S9) (ST323)	Urine	aac(6’)-ib-cr, OqxB, OqxA	NM^V^	NM	N304S	
D(UNN40_S4) (ST101)	Urine	aac(6’)-ib-cr, OqxB, OqxA,	S83Y, D87G	D553V	S438D, D475E, L495Q, Q592R, V691I	
C(UNN39_S3) (ST101)	Urine	aac(6’)-ib-cr, OqxA, OqxB				
***S*. *marcescens***^VI^						
71_S36 (SA1)	X	aac(6’)-lb-cr,	S83I, A188T, S171A, V240I	T155N, D181E, K206Q, Q696K, A636S, Q692L, I701V	T59N, R679Q, Y705H, G753S, A750V	NM
68_S34 (SA1)	Blood	oqXB				
67_S33 (SA1)	CVP Tip	oqxB				
59_S30 (SA2)	urine	aac(6’)-ib-cr, QnrS1, oqxB,	S83I, A188T	T155N, D181E, K206Q, Q696K	T59N, A592T, R679Q, Y705H, G753S, A750V	
56_S29 (SA2)	urine	aac(6’)-ib-cr, OqxB				
45_S21 (SA1)	Tracheal fluid	aac(6’)-ib-cr, oqxB	S83I, A188T, S171A, V240I	T155N, D181E, K206Q, Q696K, A636S, Q692L, I701V	T59N, R679Q, Y705H, G753S, A750V	
7_S3 (SA1)	X	oqxB,				
L(UNN48_S12) *(SA1)*	Sputum	aac(6’)-ib-cr, oqxB				
K(UNN47_S11) (SA1)	Blood	aac(6’)-ib-cr, oqxB				
G(UNN43_S7) (SA1)	Blood	aac(6’)-ib-cr, oqxB				
E(UNN41_S5) (SA1)	CVP Tip	oqxB				
B (UNN38 _S2) (SA1)	Tracheal fluid	oqxB				
***Enterobacter species* (unless otherwise stated in footnote, species is cloacae)**^VII^						
65_S32(ST436)	CVC^VIII^ Tip	oqxB, oqxA	A427S, L508Q	A155T	V402A, V582I, N746S	R378G
63_S31^IX^	Urine	aac(6’)-ib-cr, OqxA, OqxB, QnrB49	T83I, G409A, I411V	H173L, K206R, D747E	E84K, L596M, T746A	S592R
55_S28^X^	ETA^XI^	aac(6’)-ib-cr, OqxB, OqxA, QnrB49	S83Y	Same as H(UNN44_S8), I144L, S571H, D619E, N622D, A632T, I663V, E723D	L151M, F215Y, V402A, T459N, L596M, M621L, H629R, P632T, V636I, I691L, N746S	K203T, N205H, S212T, T243S, E475D, Y520H
49_S24^XII^	Urine	aac(6’)-ib-cr, QnrB66, OqxB, OqxA, QnrB9,	G409A, I411V	Same as 63_S31, A716T	NM	NM
43_S20 (ST433)^XIII^	Abdominal fluid	aac(6’)-ib-cr, OqxB, OqxA, QnrB49	S83I, I112V, L127M, A128S	I60V, Q72T, I144L, Q146T, E151Q, D159E, E161D, D359E, Q362L, N366S, D550E, S571H, 5595T, A604T, I610V, N622S, A625T, S635T, E636D, F649C, A656D, Q657G, I663V, E723D, S734A, D746E	E205D, T246N, S348T, V402A, M621L, P632A, V636I, S648T, I691L, N746A	S212H, N205H, T243S, E475D
16_S9^XIV^	urine	aac(6’)-ib-cr, QnrB1, OqxA, OqxB	NM	Same as 55_S28	Same as 55_S28, A467S	N205H, K203T, T243S, E475D, Y520H
1_S1(ST108)	CVP Tip	Aac(6’)-ib-cr, oqxB, oqxA, QnrB1	T408A, A409S, I411V, S412A, I509V, A526S, Q536R, K69IS, D709N, I737V, G759S	Same as F(UNN42_S6), T691A, I663V, D359E	F215Y, T246N, S348T, T352A, V402A, K427R, Q495L, A527S, L596M, V635I, V636I, S648A, N673S, G685S, I691L, N746K, G747D	Same as F(UNN42_S6), M273L
H(UNN44_S8)^XV^	Urine	aac(6’)-ib-cr, oqxA, oqxB, QnrB1		I60V, Q72T, Q146T, E151Q, E161D, D359E, Q362L, N366S, D550E, A625T, A632T, A656E	L151M, E205D, F215Y, S348T, T352A, V402A, Q495L, A527S, L596M, M621L, V635I, V636I, S648A, N673S, G685S, I691L, N746K, G747D	N225H, C234R, T243E, E475D, Y520H, M273L, A236E, D237K
F(UNN42_S6) (ST121)^XVI^	Urine	aac(6’)-ib-cr, QnrB1, oqxA, oqxB,	Same as 1_S1 above plus S83I, D861E	I60V, Q72T, Q362L, N366S, A625T, A632T, A696E	S80I, L151M, E205D, F215Y, S272T, S348T, T352A, V402A, K427R, Q495L, A527S, L596M, M621L, V635I, V636I, S648A, N673S, G685S, I691L, N746K, G747D, D583E	N225H, S232T, T243E, E475D, T512A, Y520H, M602V
A(UNN37_S1)^XVII^	Urine	aac(6’)-ib-cr, oqxB, oqxA, QnrS1, QnrB1	G409A, I411V	Same as 49_24	NM	NM
***C*. *freundii***^XVIII^						
51_S25 (NK^XIX^)	sputum	aac(6’)-ib-cr, QnrB49, QnrS1	T83I, N535S, N691S, N709D, G728E, E729K, E758A, S759E, G767A	H417L, A576S, A579L, A616P, V642M, R688H, A692T	S80I, E204D, N458S, E470D, F690Y	NM
48_S23 (ST63)	Catheter tip	Aac(6’)-ib-cr, QnrB2, QnrS1				
14_S7	sputum	Aac(6’)-ib-cr, QnrB38	T83I	L162M, A692T	E204D, F690Y	
***E*. *coli***^XX^						
10_S4 (ST167)	Urine	-^XXI^	S58L, D62N, E653D, S803A	D185E	S80I, E475D, P677L	I76V, S458A
***K*. *michiganensis***^XXII^						
69_S35 (ST170)	Urine	aac(6’)lb-cr, oqxB, oqxA, QnrS1	I83T	NM	S80R	NM

^III^*K*. *pneumoniae* ATCC 13883 (PRJNA244567) was used as reference strain in the comparative genome analysis

^IV^Data missing; ^V^No mutation

^VI^S. marcescens strain CDC_813–60 (ATCC 13880) (JOVM00000000/ PRJNA244355) was used as reference strain to find amino acid mutations

^VII^Unless otherwise stated in footnote, *E*. *cloacae* ATCC 13047 (CP001918.1) was used as reference strain in the comparative genomics

V^III^Central venous catheter

V^IX^ *Enterobacter asburiae*: *E*. *asburiae* L1 (CP007546.1) was used as reference strain for the comparative genomics to find amino acid mutations

^X^ *Enterobacter kobei*: *E*. *kobei* strain 35730 (JZYS01000016.1) or *E*. *cloacae* ATCC 13047 (CP001918.1) served as reference strains for amino acid mutations.

^XI^ Endotracheal aspirate

X^II^*Enterobacter asburiae*: *Enterobacter asburiae* L1 (CP007546.1) was used as reference strain for the comparative genomics to find amino acid mutations

X^III^ *Enterobacter cloacae complex “Hoffman cluster IV”*

X^IV^*Enterobacter kobei*: *E*. *kobei* strain 35730 (JZYS01000016.1) or *E*. *cloacae* ATCC 13047 (CP001918.1) served as reference strains for amino acid mutations.

^XV^*Enterobacter cloacae complex “Hoffman cluster III”*

^XVI^*Enterobacter species*

^XVII^*Enterobacter asburiae*: *Enterobacter asburiae* L1 (CP007546.1) was used as reference strain for the comparative genomics to find amino acid mutations.

^XVIII^*C*. *freundii* ATCC 8090 = MTCC 1658 (PRJNA177199) was used as reference strain for the comparative genomics to find amino acid mutations.

^XIX^MLST unknown

^XX^*Escherichia coli ATCC 25922* (CP009072) was used as subject in the comparative genomics analysis to find the amino acid mutations.

^XXI^No PMQR (plasmid-mediated quinolone resistance) gene found

^XXII^*Klebsiella michiganensis* KCTC 1686 (CP003218.1) was used as reference strain for this comparative genomic analysis to find amino acid mutations.

**Table 3:** Frequency and distribution of plasmid-mediated fluoro/-quinolone resistance (PMQR) genes in the Enterobacteriaceae isolates.

Plasmid-mediated quinolone resistance (PMQR) gene	Gene variants	Frequency (h) of occurrence per specie						Total frequency (n = 48)
		K. pneumoniae (n = 21)	S. marcescens (n = 12)	Enterobacter spp. (n = 10)	C. freundii (n = 3)	E. coli (n = 1)	K. michiganensis (n = 1)	
**Aac(6’)-Ib-cr**	-	8	7	9	3	0	1	28
**Qnr**	QnrS1	2	1	1	2	0	1	7
	QnrB1	1	0	5	0	0	0	6
	QnrB49	0	0	3	1	0	0	4
	QnrB66	1	0	1	0	0	0	2
	QnrB2	0	0	0	1	0	0	1
	QnrB9	0	0	1	1	0	0	2
	QnrB38	0	0	0	1	0	0	1
**OqxAB**	oqxA	9	0	10	0	0	1	20
	oqxB	21	11	10	0	0	1	43

No transposon or integron was found in the immediate environment of the *qnr* and *aac(6’)-Ib-cr* genes. The truncated nature of the plasmid contigs made it impossible to link the PMQR genes to particular plasmids or replicon/incompatibility types.

### 2.3 Multi locus sequence typing (MLST) and *S*. *marcescens* typing

The MLST of the isolates are shown in Table 2 and it can be seen that the isolates were very multiclonal, albeit many *K*. *pneumoniae* strains (n = 14) were of sequence type (ST) 101. The MLST of *K*. *michiganensis* was determined using that of *K*. *oxytoca* due to misidentification of the former as *K*. *oxytoca*.

Alignment of the *dnaA*, *fusA*, *gyrB*, *leuS*, *pyrG*, *rplB*, and *rpoB* housekeeping genes of all the 12 *S*. *marcescens* isolates resulted in 10 of the isolates being classified into the same clone (i.e. SA1) and two of the remaining being grouped into SA2 clone. Isolates of each clone showed no variation from the reference sequences of housekeeping genes of their respective clones. The SA2 clone only differed from SA1 clone at the nucleotide level in *dnaA* and *leuS* by a single (T106C) and double (A7G and A91G) silent single nucleotide polymorphisms (SNPs) respectively

### 2.4 Mutations in *gyrA*, *gyrB*, *parC* and *parE*

The sequences of *gyrA*, *gyrB*, *parC* and *parE* were analysed to determine the presence of mutations that could account for the high-level FQ resistance as mutations in these four genes have been incriminated in high-level FQ resistance. The mutations in *gyrA*, *gyrB*, *parC* and *parE* mostly occurred in a manner that reflected the clonality of the strains i.e. the mutations in all the genes were mostly clonally related. For instance, *K*. *pneumoniae* ST101 and ST2017 had the same mutations in all the genes except for *parC* at which only three ST101 strains (J, D and C) differed. The mutations in *K*. *pneumoniae* ST1478, ST323 and ST14 were unique. The clonal nature of the mutations was more obvious with *S*. *marcescens* in which all SA1 and all SA2 clones had the same mutations for these genes within their members. The close relation between SA1 and SA2 clones were further underscored by the similarity and few differences in the mutations observed in their *gyrA*, *gyrB*, *parC* and *parE* genes. This observation corroborated the correctness of our typing system. Within the *Enterobacter spp*. and *C*. *freundii* isolates, the uniqueness of their *gyrA*, *gyrB*, *parC* and *parE* mutations was but a reflection of their different species or clones. However, *C*. *freundii* isolates 51_S25 and 48_S23, except isolate 14_S7, had same mutations in *gyrB*, *parC* and *parE*. Positions 83 in *gyrA* was a common mutation position in many isolates and species whilst mutations at position 80 in *parC* was common in *C*. *freundi*, *E*. *coli*, *Enterobacter spp*,. *K*. *pneumoniae* and *K*. *michiganensis*. No *parE* mutations were found in *K*. *pneumoniae* (except in 47_S22), *S*. *marcescens*, *C*. *freundii* and *K*. *michiganensis* (Table 2).

## 3. Discussion

The importance of this study is underscored by the discovery that there is a substantial number of high-level fluoroquinolone resistance among clonal and multiclonal Enterobacteriaceae circulating in hospitals in Durban, South Africa. This is evinced by the exceptionally high-level of MICs, mostly from 64mg/L to >512mg/L that was recorded among isolates of all species (Table 1 and S3 Table). To our knowledge, no study in South Africa or Africa has discovered such a number of high-level FQ-resistant Enterobacteriaceae with multiple mechanisms of FQ resistance, albeit not all the collected 82 isolates were analysed. This is a rather disturbing observation given the importance of FQ in the management of several bacterial infections in South Africa and the importance of South Africa as a destination for many medical tourists from Africa and Europe [1,4].

To determine the role of efflux pumps as mechanisms underlying this high-level FQ resistance, the isolates were grown in the presence of sub-MICs of CCCP, RSP and VRP. Interestingly, CCCP, a protonophore that indirectly reduce efflux pumps activity by reducing ATP production and unbalancing the electrochemical gradient could not significantly affect the isolates’ MICs. Gram-negative bacteria, including Enterobacteriaceae, are known to have ABC-type efflux pumps that are directly powered/energised by ATP hydrolysis and secondary transporters (sodium-proton symporters) such a MATE-, SMR-, MFS- and RND-type efflux pumps that are powered by the proton motive force (PMF) [16–18]. Hence, the inability of CCCP to reduce the FQ MICs is rather surprising but it shows that efflux pumps affected by CCCP were not involved in resistance to CIP, NOR and NAL.

VRP has been shown recently to affect MATE-type efflux pumps by directly blocking the pumps channels [17,15]. Subsequently, we are convinced that MATE-type efflux pumps were involved in conferring resistance to CIP (n = 5) and NOR (n = 6) in a few of the isolates affected by VRP (Table 1). Moreover, it can be concluded that RSP, which is believed to affect RND-type efflux pumps in Gram-negative bacteria [15,19,20], was mostly involved in reducing the MICs of CIP (n = 16) and NOR (n = 24) significantly in many of the isolates. Nevertheless, it must be borne in mind that in all a total of 25 isolates, which is approximately half of the total sample size, were affected by the inhibitors, suggesting that efflux as a mechanism of FQ resistance, was present in one out of every two isolates.

This implicating of efflux as a FQRM in approximately half of the isolates is comparable to five studies involving FQ resistance, three of which involved Salmonella [9,10,21], one involved *Vibrio cholerae* [13]and the last involved *Aeromonas veronii* and *A*. *hydrophila* [12]. Out of these five studies, efflux was implicated as a FQRM in three [9,10,13] whilst they were found not to be involved in two studies [12,21]. Thus, efflux is not always a mechanism of resistance to FQ, albeit an important one. That the inhibitors were unable to reverse FQ resistance in any of the isolates is a testimony that other mechanisms other than efflux was involved [21].

Annotation of the WGS data showed that the most predominant PMQR was *oqxAB*, which has never been reported as a FQRM in any study in South Africa or Africa as far as we are concerned [1]. This is possibly because PCR has been used in gene-targeted amplification and sequencing, which has not involved the search for *oqxAB* genes; this stresses the need to incorporate WGS into antibiotic resistance research in Africa [1]. Interestingly, *oqxA* were not as prevalent as *oqxB* in the isolates and a pattern or correlation could not be established between their presence and the MICs of the isolates. However, as suggested by other authors [22,23], we also hypothesise that oqxB can work through *acrA* in the absence of *oqxA* as *oqxAB* has been shown to be a supplementary resistance mechanism to *acrAB* and its increased expression is dependent on a functional *acrAB* [22,23]. Hence, oqxAB is adding up to other mechanisms to increase the FQ resistance in the isolates and cannot be a major mechanism, particularly when equally or much higher MICs were recorded in strains (e.g. *E*. *coli* and *C*. *freundii* vis-à-vis *S*. *marcescens* and *Enterobacter spp*.) in which they were absent (Table 2).

The second most dominant PMQR gene was *aac-(6’)-Ib-cr*, which makes this the first study to report on the presence of this gene as a FQRM in Enterobacteriaceae in South Africa. However, this gene has been implicated in FQ resistance in *E*. *coli* in Nigeria and a few other African countries [1]. The *qnr* genes were the least prevalent of the PMQR genes although they are the only PMQR genes to have been reported already in S. Africa and are commonly identified in other African countries [1,12,21]. The commonest variants of the *qnr* gene identified in this study were the *qnrS1*, *qnrB1* and *qnrB49*, which agrees with a study by Smith et al. [10] involving Salmonella and by Chenia [12] involving *A*. *veronii* and *A*. *hydrophila* that found only *qnrS1* and *qnrB* and *qnrS* respectively. However, Chenia found a higher percentage of *qnrB* (41%) than *qnrS* (24%) from freshwater fish whilst we found *qnrS*1 to be higher than *qnrB* in clinical samples. There is the need to undertake further surveillance and molecular epidemiology to assess the possibility of these *qnr* genes being transferred from freshwater foods to humans or of human activities being the source of these resistance genes in freshwater and the environment as has been recently reported in China where PMQR genes in high numbers were found in *Aeromonas spp*. from rivers contaminated by hospital and aquaculture effluents [2,12].

On the whole, more studies have implicated mutations in *gyrA*, *gyrB*, *parC* and *parE* as the major mechanism of FQ resistance as the PMQR genes only mediate low-level FQ resistance [9–11,13]. This can be seen in our findings in which mutations in these four genes (except in *parE* in most isolates) were found in all isolates as compared to the PMQR genes that were not found in all strains. The numerous nature of the mutations observed in the individual genes as well as collectively in all four genes will surely add up to make the strains highly resistant as recorded. The common mutations seen per clone testifies to the clonality of the isolates and authenticates our typing scheme, particularly in *S*. *marcescens*. One of the commonest *gyrA* mutation recorded in literature is Ser83Ile or Ser83Tyr, which was also found in our strains (Table 2). Chenia [12] found Ser80Ile in the *parC* of her *A*. *hydrophila/veronii* strains and this was also found among our isolates. As the mechanism of action of FQ involves the DNA gyrase and topoisomerase IV, it is reasonable that mutations in genes encoding these FQ targets should mediate high-level FQ resistance [1,2].

Hence, we conclude that high-level FQ resistance in Enterobacteriaceae in South Africa is mainly mediated by numerous and diverse mutations in *gyrA*, *gyrB*, *parC* and *parE*, in synergy with efflux upregulation and PMQR genes, and that these highly resistant strains are being disseminated vertically by clonal and multiclonal expansion and horizontally via plasmids in hospitals. Given that the use of other antibiotics can select for PMQR genes [8], the need for antibiotic stewardship is exceptionally important to prevent further escalation of this menace.

## 4. Materials and methods

### 4.1 Ethical approval

The Biomedical Research Ethics Committee of the University of KwaZulu-Natal approved this study under the reference number BE040/14.

### 4.2 Bacterial strains

From a collection of 82 Enterobacteriaceae isolates with reduced susceptibility to FQ, which were collected from a private pathology laboratory in Durban, South Africa between 2012 and 2013, 48t isolates comprising of *Klebsiella pneumoniae* (n = 21), *Serratia marcescens* (n = 12), *Enterobacter spp*. (n = 10), *Citrobacter freundii* (n = 3), *Klebsiella michiganensis* (n = 1), and *Escherichia coli* (n = 1) that were fully FQ-resistant per further micro-broth dilution (MIC) testing, were selected. Disc diffusion with ciprofloxacin (CIP), norfloxacin (NOR), and nalidixic acid (NAL) was used to identify and collate these 82 isolates in the pathology laboratory, where all isolates with reduced susceptibility to FQ were included; however, further confirmatory testing with MIC showed that only 48 were fully FQ-resistant per EUCAST (2016) breakpoints [14,24]. Hence, only the 48 isolates were included. The isolates were presented by 10 different hospitals to the private pathology laboratory and were obtained from patients of both sex between the ages of 2 months and 83 years. *E*. *coli* ATCC 25922 and *K*. *oxytoca ATCC 13178* were used as controls in the antibiotic sensitivity testing/screening (S2 Table).

Identification of the isolates were initially done with Vitek II and confirmed by NCBI BLAST and NCBI’s ANI report of the whole genome sequence of the isolates. Thus, the *Enterobacter spp*. were identified as *Enterobacter cloacae* (n = 2), *E*. *asburiae* (n = 3), *E*. *kobei* (n = 2), *Enterobacter cloacae complex “Hoffman cluster IV”* (n = 1), *Enterobacter cloacae complex “Hoffman cluster III”* (n = 1), and *Enterobacter sp*. (n = 1).

### 4.3 Minimum inhibitory concentrations (MICs) of CIP, NOR, NAL in the absence and presence of CCCP, reserpine (RSP) and verapamil (VRP): Evaluating the effect of efflux on fluoroquinolone resistance

Pure powders of CIP, NOR, NAL, carbonyl cyanide 3-chlorophenylhydrazone (CCCP), reserpine (RSP) and verapamil (VRP) were purchased from Sigma Aldrich (St. Louis, MO, USA) and used for the broth micro dilution assays. MIC determination and results interpretation for CIP, NOR and NAL were done according to EUCAST guidelines and breakpoints (2016) [14]. Deionized water was used to make VRP solutions whilst RSP was prepared in dimethyl sulfoxide (DMSO) and CCCP in 50% methanol (v/v) [25]. Cation-adjusted Mueller Hinton broth was used to determine the minimum inhibitory concentrations (MICs) of CIP, NOR, NAL, and VRP. All solutions were prepared on the day of the experiment and kept protected from the light.

In determining the effect of CCCP, RSP and VRP on CIP, NOR and NAL MICs, a sub-MIC (i.e. 0.5 × MIC) of CCCP, RSP and VRP viz., 8, 256 and 256mg/L were used respectively whilst serially increasing the concentrations of the antibiotics to determine the change in MICs (Table 1 and S1 Table). A sub-MIC was used to reduce the possibility of CCCP, RSP and VRP killing the cells and interfering with the true MIC of the antibiotic-inhibitor combination. CCCP was used as a protonophore to assess its effects on the antibiotics’ MICs through a reduction in efflux activity and disruption of the transmembrane electrochemical potential/gradient. It acts by unbalancing the transmembrane proton gradient, reducing ATP production and indirectly inhibiting efflux activity [16]. RSP and VRP are broad spectrum efflux inhibitors that are commonly used in efflux inhibition experiments. They were used in this study to assess their effect on the antibiotics’ MIC through inhibition of RND and MATE efflux pumps respectively [16,17,19,20].

### 4.4 Genomic DNA extraction, library preparation and whole genome sequencing

Genomic DNA (gDNA) of the isolates were extracted using the GenElute Bacterial Genomic DNA kit (Sigma Aldrich, St. Louis, MO, USA) per the manufacturer’s instructions. The Qubit and Nanodrop were used to determine the concentrations and purity of extracted gDNA. The quality and integrity of the purified gDNA were confirmed by performing gel electrophoresis before proceeding to library generation. Libraries were quantified on the Bioanalyzer (Agilent Technologies) and combined in an equimolar mixture. A nanogram of gDNA was used as input for the Nextera XT kit (Illumina) to generate 300 bp paired-end libraries followed by sequencing on an Illumina MiSeq platform; a genome coverage of 20-90x was generated for all the isolates [6,26,27] (S3 Table).

### 4.4 Genomic sequencing analysis

Raw sequence reads of the isolates were adaptor- and quality-trimmed using Trimmomatic [28] and deposited at sequence read archive (SRA) under project number PRJNA287968. The raw reads were assembled with SPAdes 3.9 (https://cge.cbs.dtu.dk/services/SPAdes/) and contigs smaller than 200 bps were removed. The resulting fasta files were deposited at Genbank under the bioproject PRJNA287968. Annotation of the whole genome to determine plasmid-mediated fluoroquinolone resistance (PMQR) genes and chromosomal-borne *gyrA*, *gyrB*, *parC* and *parE* genes was done with ResFinder (https://cge.cbs.dtu.dk/services/ResFinder/) and Prokaryotic Genome Annotation Pipeline (PGAP) (https://www.ncbi.nlm.nih.gov/genome/annotation_prok/) respectively [6,29].

### 4.5 Bioinformatic analysis

MLST of the isolates were determined using MLST 1.7 pipeline at the Center for Genomic Epidemiology (https://cge.cbs.dtu.dk/services/MLST/). The PMQR genes (*aac(6’)-Ib-cr*, *qepA*, *qnr* and *oqxAB*) were tabulated and their genetic support/environment, namely transposons and integrons, were searched for using the annotations provided by PGAP. Translated nucleotide Basic Local Alignment Search Tool [tBLASTn] was used to search for genetic elements in the immediate environment of the *aac(6’)-Ib-cr*, and *qnr* genes.

Typing of the *S*. *marcescens* isolates was done in-house using seven house-keeping genes that had been used for typing *Enterobacter cloacae*.: *dnaA*, *fusA*, *gyrB*, *leuS*, *pyrG*, *rplB*, and *rpoB* [30]. The same housekeeping genes in each *S*. *marcescens* isolate were aligned to each other in a gene-gene format using nucleotide BLAST [BLASTn] and SNPs were called for all seven genes. A single SNP in any of the genes was used as a cut-off for categorising the isolates into different clones.

Mutations in the chromosomal-borne *gyrA*, *gyrB*, *parC* and *parE* genes were determined using tBLASTn to call SNPs in these genes. Fluoro/-quinolone susceptible reference/type strains that were used for each species were as follows: *K*. *pneumoniae* ATCC 13883 (PRJNA244567) for *K*. *pneumoniae*; *S*. *marcescens* strain CDC_813–60 (ATCC 13880) (JOVM00000000/ PRJNA244355) for *S*. *marcescens; E*. *cloacae* ATCC 13047 (CP001918.1) for all *Enterobacter spp*. except *E*. *asburiae* and *gyrA* in *E*. *kobei*; *Enterobacter asburiae* L1 (CP007546.1) for *E*. *asburiae*; *E*. *kobei* strain 35730 (JZYS01000016.1) or *E*. *cloacae* ATCC 13047 (CP001918.1) for *E*. *kobei*; *C*. *freundii* ATCC 8090 = MTCC 1658 (PRJNA177199) for *C*. *freundii*; *E*. *coli ATCC 25922* (CP009072) *E*. *coli*; *Klebsiella michiganensis* KCTC 1686 (CP003218.1) for *K*. *michiganensis*. For *gyrB*, *parC* and *parE* in *E*. *kobei*, *E*. *cloacae* type strain was used as the reference strain as the *E*. *kobei’s* reference strain returned no *gyrB*, *parC* and *parE* genes when it was called/searched with BLAST+.

### 4.6 Data analysis

An MIC fold change was defined as the ratio of the MIC of antibiotic and inhibitor to that of the antibiotic alone. A fold change of ≥8 was deemed as significant and indicative of efflux activity [15]. Where the MIC was >512, an absolute value of 512 was used, specifically for NAL (S1 Table). The frequency and distribution of the PMQR genes per species were tabulated and translated into a graph (Fig 1).

## Supporting information

S1 Table MICs of inhibitors alone and of nalidixic acid (NAL) MIC changes upon adding carbonyl cyanide-m-chlorophenylhydrazine (CCCP), verapamil (VRP) and reserpine (RSP). (DOC)

S2 Table Antimicrobial susceptibility (disc diffusion) results for ciprofloxacin, norfloxacin and nalidixic acid on the Enterobacteriaceae isolates. (DOC)

S3 Table Genomic features of the sequenced Enterobacteriaceae isolates. (DOC)
