# Supplementary material for: Genomic and phenotypic characterisation of fluoroquinolone resistance mechanisms in Enterobacteriaceae in Durban, South Africa
Source: PLoS One. 2017 Jun 21;12(6):e0178888. doi: 10.1371/journal.pone.0178888 (PMC5479536; doi:10.1371/journal.pone.0178888)
Supplement: S1 Table — (DOC) [file pone.0178888.s001.doc]

# **Genomic and Phenotypic Characterisation of Fluoroquinolone Resistance Mechanisms in Enterobacteriaceae in South Africa.**

John Osei Sekyere^1*^ Daniel Gyamfi Amoako^2,3^

**S1 Table. MICs of inhibitors alone and of nalidixic acid (NAL) MIC changes upon adding carbonyl cyanide-m-c**hlorophenylhydrazine (CCCP), verapamil (VRP) and reserpine (RSP).

| Isolate | MIC of inhibitors (mg/L) | | | MIC of Nalidixic acid (NAL) (mg/L) | | | |
| --- | --- | --- | --- | --- | --- | --- | --- |
|  | CCCP | VRP | RSP | NAL | NAL + CCCP | NAL + VRP | NAL + RSP (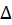) |
| *E. coli ATCC 25922* | 8 | >512 | >512 | **2** | 2 | 2 | 2 |
| *K. oxytoca ATCC 13178* | 8 | >512 | >512 | **8** | 8 | 4 | 8 |
| ***K. pneumoniae*** | | | | | | | |
| C(UNN39_S3) | 64 | >512 | >512 | **>512** | >512 | >512 | >512 |
| D(UNN40_S4) | 32 | >512 | >512 | **>512** | >512 | >512 | >512 |
| I(UNN45_S9) | 16 | >512 | >512 | **>512** | >512 | >512 | 512 |
| J(UNN46_S10) | 32 | >512 | >512 | **>512** | >512 | >512 | 512 |
| 3_S2 | 32 | >512 | >512 | **>512** | >512 | >512 | 512 |
| 12_S5 | 32 | >512 | >512 | **>512** | >512 | >512 | >512 |
| 13_S6 | 64 | >512 | >512 | **>512** | >512 | >512 | 512 |
| 15_S8 | 16 | >512 | >512 | **>512** | >512 | >512 | >512 |
| 18_S10 | 64 | >512 | >512 | **>512** | >512 | >512 | 512 |
| 20_S11 | 32 | >512 | >512 | **>512** | >512 | >512 | >512 |
| 21_S12 | 32 | >512 | >512 | **>512** | >512 | >512 | 512 |
| 29_S13 | 16 | >512 | >512 | **>512** | >512 | >512 | 512 |
| 30_S14 | 32 | >512 | >512 | **>512** | >512 | >512 | 512 |
| 32_S15 | 16 | >512 | >512 | **>512** | >512 | >512 | >512 |
| 34_S16 | 64 | >512 | >512 | **>512** | >512 | >512 | 512 |
| 35_S17 | 16 | >512 | >512 | **>512** | >512 | >512 | 512 |
| 36_S18 | 64 | >512 | >512 | **>512** | >512 | >512 | 512 |
| 38_S19 | 32 | >512 | >512 | **>512** | >512 | >512 | >512 |
| 47_S22 | 16 | >512 | >512 | **>512** | >512 | >512 | 512 |
| 52_S26 | 32 | >512 | >512 | **>512** | >512 | >512 | 512 |
| 53_S27 | 32 | >512 | >512 | **>512** | >512 | >512 | 512 |
| ***S. marcescens*** | | | | | | | |
| B(UNN38 _S2) | 256 | >512 | >512 | **>512** | >512 | >512 | 512 |
| E(UNN41_S5) | 256 | >512 | >512 | **>512** | >512 | >512 | 512 |
| G(UNN43_S7) | 256 | >512 | >512 | **>512** | >512 | >512 | 512 |
| K(UNN47_S11) | 128 | >512 | >512 | **>512** | >512 | >512 | 512 |
| L(UNN48_S12) | 64 | >512 | >512 | **>512** | >512 | >512 | 512 |
| 7_S3 | 64 | >512 | >512 | **>512** | >512 | 512 | 256 (2) |
| 45_S21 | 128 | >512 | >512 | **>512** | >512 | >512 | 512 |
| 56_S29 | 16 | >512 | >512 | **>512** | >512 | >512 | 512 |
| 59_S30 | 128 | >512 | >512 | **>512** | >512 | >512 | 512 |
| 67_S33 | 32 | >512 | >512 | **>512** | >512 | 512 | 256 (2) |
| 68_S34 | 256 | >512 | >512 | **>512** | >512 | >512 | 512 |
| 71_S36 | 128 | >512 | >512 | **>512** | >512 | >512 | 512 |
| ***Enterobacter species*** | | | | | | | |
| A (UNN37_S1) | 16 | >512 | >512 | **>512** | >512 | >512 | 512 |
| F (UNN42_S6) | 64 | >512 | >512 | **>512** | >512 | >512 | 512 |
| H (UNN44_S8) | 32 | >512 | >512 | **>512** | >512 | >512 | 256 |
| 1_S1 | 64 | >512 | >512 | **>512** | >512 | >512 | 128 (4) |
| 16_S9 | 32 | >512 | >512 | **>512** | >512 | >512 | 512 |
| 43_S20 | 64 | >512 | >512 | **>512** | >512 | >512 | 128 (4) |
| 49_S24 | 64 | >512 | >512 | **>512** | >512 | >512 | 512 |
| 55_S28 | 64 | >512 | >512 | **>512** | >512 | >512 | 512 |
| 63_S31 | 32 | >512 | >512 | **>512** | >512 | >512 | 128 (4) |
| 65_S32 | 64 | >512 | >512 | **>512** | >512 | >512 | 512 |
| ***E. coli*** | | | | | | | |
| 10_S4 | 128 | >512 | >512 | **>512** | >512 | >512 | 512 |
| ***C. freundii*** | | | | | | | |
| 14_S7 | 128 | >512 | >512 | **>512** | >512 | 512 | 256 (2) |
| 48_S23 | 256 | >512 | >512 | **>512** | >512 | 512 | 512 |
| 51_25 | 128 | >512 | >512 | **>512** | >512 | >512 | 512 |
| ***K. michiganensis*** | | | | | | | |
| 69_S35 | 64 | >512 | >512 | **>512** | >512 | >512 | 256 (2) |
