# Supplementary material for: Genomic and phenotypic characterisation of fluoroquinolone resistance mechanisms in Enterobacteriaceae in Durban, South Africa
Source: PLoS One. 2017 Jun 21;12(6):e0178888. doi: 10.1371/journal.pone.0178888 (PMC5479536; doi:10.1371/journal.pone.0178888)
Supplement: S2 Table — (DOC) [file pone.0178888.s002.doc]

# **Genomic and Phenotypic Characterisation of Fluoroquinolone Resistance Mechanisms in Enterobacteriaceae in South Africa.**

John Osei Sekyere^1*^ Daniel Gyamfi Amoako^2,3^

**S2 Table. Antimicrobial susceptibility (disc diffusion) results for ciprofloxacin, norfloxacin and nalidixic acid on the Enterobacteriaceae isolates**

| Isolate | Ciprofloxacin[[1]](#footnote-2) [5 µg], mm | Norfloxacin^1^ [10 µg], mm | Nalidixic acid [30 µg], mm |
| --- | --- | --- | --- |
| *E. coli* ATCC 25922 | 32 | 31 | 24 |
| *S.aureus ATCC 29213* | 25 | 26 | 12 |
| ***K. pneumoniae*** |  |  |  |
| C | 0 | 0 | 0 |
| D | 0 | 0 | 0 |
| I | 19 | 18 | 0 |
| J | 0 | 0 | 0 |
| 3 | 0 | 0 | 0 |
| 12 | 0 | 0 | 0 |
| 13 | 0 | 0 | 0 |
| 15 | 0 | 0 | 0 |
| 18 | 0 | 0 | 0 |
| 20 | 0 | 0 | 0 |
| 21 | 0 | 0 | 0 |
| 29 | 0 | 0 | 0 |
| 30 | 0 | 0 | 0 |
| 32 | 0 | 0 | 0 |
| 34 | 0 | 0 | 0 |
| 35 | 0 | 0 | 0 |
| 36 | 0 | 0 | 0 |
| 38 | 0 | 0 | 0 |
| 47 | 0 | 0 | 0 |
| 52 | 0 | 0 | 0 |
| 53 | 0 | 0 | 0 |
| ***S. marcescens*** | | | |
| B | 0 | 0 | 0 |
| E | 0 | 0 | 0 |
| G | 0 | 0 | 0 |
| K | 0 | 0 | 0 |
| L | 0 | 0 | 0 |
| 7 | 0 | 0 | 0 |
| 45 | 0 | 0 | 0 |
| 56 | 0 | 0 | 0 |
| 59 | 0 | 0 | 0 |
| 67 | 0 | 0 | 0 |
| 68 | 0 | 0 | 0 |
| 71 | 0 | 0 | 0 |
| ***E. cloacae*** | | | |
| A | 0 | 0 | 0 |
| F | 0 | 0 | 0 |
| H | 0 | 0 | 0 |
| 1 | 0 | 0 | 0 |
| 16 | 0 | 0 | 0 |
| 43 | 0 | 0 | 0 |
| 49 | 0 | 0 | 0 |
| 55 | 0 | 0 | 0 |
| 63 | 8 | 0 | 0 |
| 65 | 0 | 0 | 0 |
| ***E. coli*** | | | |
| 10 | 0 | 0 | 0 |
| ***C. freundii*** | | | |
| 48 | 0 | 0 | 0 |
| 51 | 0 | 0 | 0 |
| ***K. michiganensis*** | | | |
| 69 | 0 | 0 | 0 |

1. Resistance < 19mm [↑](#footnote-ref-2)
