# Supplementary material for: Genomic and phenotypic characterisation of fluoroquinolone resistance mechanisms in Enterobacteriaceae in Durban, South Africa
Source: PLoS One. 2017 Jun 21;12(6):e0178888. doi: 10.1371/journal.pone.0178888 (PMC5479536; doi:10.1371/journal.pone.0178888)
Supplement: S3 Table — (DOC) [file pone.0178888.s003.doc]

# **Genomic and Phenotypic Characterisation of Fluoroquinolone Resistance Mechanisms in Enterobacteriaceae in South Africa.**

John Osei Sekyere^1*^ Daniel Gyamfi Amoako^2,3^

**S3 Table. Genomic features of the sequenced Enterobacteriaceae isolates.**

| Isolate | Species | Contigs | Size (Mb) | G+C | Number of RNAs | Number of Coding sequences |
| --- | --- | --- | --- | --- | --- | --- |
| C(UNN39_S3) | *K. pneumoniae* | 88 | 5.5 | 57.2 | 80 | 5342 |
| D(UNN40_S4) | *K. pneumoniae* | 186 | 5.8 | 56.9 | 100 | 5502 |
| I(UNN45_S9) | *K. pneumoniae* | 118 | 5.7 | 57 | 96 | 5431 |
| J(UNN46_S10) | *K. pneumoniae* | 133 | 5.7 | 57.0 | 81 | 5743 |
| 3_S2 | *K. pneumoniae* | 220 | 5.8 | 56.8 | 76 | 5709 |
| 12_S5 | *K. pneumoniae* | 450 | 5.8 | 57.0 | 82 | 5947 |
| 13_S6 | *K. pneumoniae* | 413 | 5.8 | 56.9 | 84 | 5917 |
| 15_S8 | *K. pneumoniae* | 442 | 5.9 | 51.8 | 88 | 6110 |
| 18_S10 | *K. pneumoniae* | 388 | 5.8 | 56.9 | 81 | 5914 |
| 20_S11 | *K. pneumoniae* | 378 | 6.0 | 56.9 | 93 | 6056 |
| 21_S12 | *K. pneumoniae* | 489 | 6.1 | 56.8 | 85 | 6277 |
| 29_S13 | *K. pneumoniae* | 259 | 5.9 | 57.0 | 82 | 5991 |
| 30_S14 | *K. pneumoniae* | 365 | 5.9 | 56.9 | 78 | 5927 |
| 32_S15 | *K. pneumoniae* | 295 | 5.7 | 57.1 | 92 | 5794 |
| 34_S16 | *K. pneumoniae* | 347 | 5.9 | 56.9 | 81 | 5946 |
| 35_S17 | *K. pneumoniae* | 397 | 5.9 | 56.9 | 86 | 5988 |
| 36_S18 | *K. pneumoniae* | 325 | 5.9 | 56.9 | 73 | 5891 |
| 38_S19 | *K. pneumoniae* | 611 | 6.0 | 56.7 | 97 | 6095 |
| 52_S26 | *K. pneumoniae* | 371 | 5.9 | 56.8 | 72 | 5506 |
| 53_S27 | *K. pneumoniae* | 423 | 5.8 | 57.0 | 99 | 5890 |
| B (UNN38_S2) | *S. marcescens* | 263 | 6.3 | 58.6 | 87 | 5987 |
| E(UNN41_S5) | *S. marcescens* | 304 | 6.3 | 58.5 | 84 | 6583 |
| G(UNN43_S7) | *S. marcescens* | 282 | 6.3 | 58.5 | 86 | 6483 |
| K(UNN47_S11) | *S. marcescens* | 341 | 5.8 | 57.2 | 83 | 5875 |
| L(UNN48_S12) | *S. marcescens* | 294 | 6.3 | 58.6 | 91 | 6071 |
| 7_S3 | *S. marcescens* | 606 | 6.4 | 58.4 | 94 | 6759 |
| 45_S21 | *S. marcescens* | 535 | 6.2 | 58.7 | 95 | 6532 |
| 56_S29 | *S. marcescens* | 527 | 5.8 | 58.8 | 106 | 5997 |
| 59_S30 | *S. marcescens* | 485 | 5.9 | 58.7 | 98 | 6130 |
| 67_S33 | *S. marcescens* | 458 | 6.4 | 58.5 | 90 | 6703 |
| 68_S34 | *S. marcescens* | 484 | 6.4 | 58.5 | 82 | 6721 |
| 71_S36 | *S. marcescens* | 682 | 6.4 | 58.5 | 93 | 6661 |
| A (UNN37_S1) | *E. asburiae* | 161 | 5.0 | 54.8 | 80 | 4671 |
| H (UNN44_S8) | *Enterobacter cloacae complex “Hoffman cluster III”* | 101 | 4.8 | 55.6 | 75 | 4597 |
| F (UNN42_S6) | *E. cloacae* | 280 | 6.3 | 58.6 | 89 | 6051 |
| 1_S1 | *E. cloacae* | 230 | 5.2 | 54.8 | 82 | 5144 |
| 16_S9 | *E. kobei* | 350 | 5.5 | 54.3 | 81 | 5628 |
| 43_S20 | *Enterobacter cloacae complex “Hoffman cluster IV”* | 586 | 5.4 | 55.1 | 82 | 5602 |
| 49_S24 | *E. asburiae* | 955 | 5.5 | 55.2 | 98 | 5097 |
| 55_S28 | *E. kobei* | 524 | 5.5 | 54.3 | 94 | 5626 |
| 63_S31 | *E. asburiae* | 460 | 5.6 | 55.2 | 88 | 5796 |
| 65_S32 | *E. cloacae* | 474 | 5.2 | 54.5 | 94 | 5290 |
| 10_S4 | *E. coli* | 739 | 5.2 | 50.7 | 103 | 5508 |
| 14_S7 | *C. freundii* | 442 | 5.9 | 51.8 | 88 | 6110 |
| 48_S23 | *C. freundii* | 278 | 4.9 | 51.9 | 69 | 4976 |
| 51_25 | *C. freundii* | 379 | 5.6 | 51.9 | 73 | 5386 |
| 69_S35 | *K. michangenesis* | 67 | 1.5 | 55.6 | 33 | 1392 |
